# Literature-Based Discovery Predicts Antihistamines Are a Promising Repurposed Adjuvant Therapy for Parkinson’s Disease

**DOI:** 10.3390/ijms241512339

**Published:** 2023-08-02

**Authors:** Gabriella Tandra, Amy Yoone, Rhea Mathew, Minzhi Wang, Chadwick M. Hales, Cassie S. Mitchell

**Affiliations:** 1 Laboratory for Pathology Dynamics, Georgia Institute of Technology, Atlanta, GA 30332, USA; 2 Neural Engineering Center, Georgia Institute of Technology, Atlanta, GA 30332, USA; 3 Department of Biomedical Engineering, Georgia Institute of Technology, Emory University, Atlanta, GA 30332, USA; 4 College of Computing, Georgia Institute of Technology, Atlanta, GA 30332, USA; 5 Department of Neurology and Center for Neurodegenerative Disease, Emory University School of Medicine, Atlanta, GA 30322, USA; cmhales@emory.edu; 6 Machine Learning Center at Georgia Tech, Georgia Institute of Technology, Atlanta, GA 30332, USA

**Keywords:** Parkinson’s disease, antihistamines, repurposed drugs, machine learning, artificial intelligence, movement disorders

## Abstract

Parkinson’s disease (PD) is a movement disorder caused by a dopamine deficit in the brain. Current therapies primarily focus on dopamine modulators or replacements, such as levodopa. Although dopamine replacement can help alleviate PD symptoms, therapies targeting the underlying neurodegenerative process are limited. The study objective was to use artificial intelligence to rank the most promising repurposed drug candidates for PD. Natural language processing (NLP) techniques were used to extract text relationships from 33+ million biomedical journal articles from PubMed and map relationships between genes, proteins, drugs, diseases, etc., into a knowledge graph. Cross-domain text mining, hub network analysis, and unsupervised learning rank aggregation were performed in SemNet 2.0 to predict the most relevant drug candidates to levodopa and PD using relevance-based HeteSim scores. The top predicted adjuvant PD therapies included ebastine, an antihistamine for perennial allergic rhinitis; levocetirizine, another antihistamine; vancomycin, a powerful antibiotic; captopril, an angiotensin-converting enzyme (ACE) inhibitor; and neramexane, an N-methyl-D-aspartate (NMDA) receptor agonist. Cross-domain text mining predicted that antihistamines exhibit the capacity to synergistically alleviate Parkinsonian symptoms when used with dopamine modulators like levodopa or levodopa–carbidopa. The relationship patterns among the identified adjuvant candidates suggest that the likely therapeutic mechanism(s) of action of antihistamines for combatting the multi-factorial PD pathology include counteracting oxidative stress, amending the balance of neurotransmitters, and decreasing the proliferation of inflammatory mediators. Finally, cross-domain text mining interestingly predicted a strong relationship between PD and liver disease.

## 1. Introduction

Parkinson’s disease (PD) is a common movement disorder in those over the age of 60 years caused by a dopamine deficit, which leads to an imbalance of motor, cognitive, and emotional loops in the basal ganglia circuitry of the brain [1]. The loss of dopaminergic neurons in the substantia nigra and impairment of nerve cells in the basal ganglia contribute to the dopamine deficit within the brain [2]. Therefore, many treatments target dopamine production to counteract this deficit.

The common symptoms of PD include akinesia in combination with either tremor at rest or rigidity. There is currently no treatment to stop the progression of the disease. However, there are treatments such as physiotherapies, medications, and surgeries to help relieve the symptoms of PD. Many patients take a medication called L-Dihydroxyphenylalanine (levodopa), a dopamine precursor that is widely used in the systematic treatment of PD. Levodopa is highly effective in reducing motor symptoms because it can cross the blood–brain barrier to be metabolized into dopamine [3]. However, as the storage capacity of the central nervous system for levodopa and dopamine declines, the efficacy of levodopa decreases. The medication demonstrates a “wearing-off” phenomenon due to dopamine fluctuation throughout the day. This effect entails motor complications each day as the medicine wears off [4]. The “wearing off” phenomenon of levodopa has inspired many studies to look for new ways to delay or ameliorate motor complications by either prescribing drugs before the use of, or in combination with, levodopa. One such example is the levodopa-carbidopa.

Likewise, this study seeks to identify repurposed drugs that could be used as adjuvant therapies to further improve PD symptoms when used in combination with dopamine replacement and/or other standard-of-care therapies. Drug repurposing is the identification of new uses for approved or investigational drugs. Repurposed drugs have already been tested in humans. As such, information on their pharmacology, formulation, and potential toxicity is available. Repurposing builds upon previous research and development efforts, which reduces overall development costs and timelines. Furthermore, new targets and pathways can be revealed and exploited [5]. Examples of successful drug repurposing include raloxifene, originally used for osteoporosis but now utilized for breast cancer treatment [6], and the sedative thalidomide, which is now used to treat myeloma [7].

The objective of this study was to use artificial intelligence to rank the most promising repurposed drug candidates for PD using text relationships extracted from 33+ million biomedical journal articles contained within PubMed. Natural language processing (NLP) techniques are used to extract text from journal articles and map relationships between genes, proteins, drugs, diseases, etc., into a network called a “knowledge graph”. SemNet 2.0 [8,9] is a recently developed software that queries a biomedical knowledge graph and utilizes unsupervised machine learning to determine the most “relevant” or important concepts to user-specified targets.

This study evaluates existing pharmacologic substances in the knowledge graph that a modified unsupervised learning rank aggregation algorithm deems [8] as most relevant to PD and levodopa. Furthermore, a new form of hub network analysis for cross-domain text mining [10] is performed. Hub analysis enables the inclusion of more distant or under-represented literature relationships. Examples include relationships to more distant domains (such as those outside of neurology), lesser-studied relationships, or novel relationships that presently have fewer citations for their support. Aggregated analysis and validatory literature review is then performed to better understand why the artificial intelligence algorithm recommends specific drugs as beneficial adjuvant therapies for PD.

The benefits of drug repurposing using cross-domain text mining and artificial intelligence are as follows: (1) it allows multiple types of relationships to be mapped across domains, such as ontology, neurology, cardiology, etc.; (2) it removes human biases for ranking and prioritizing promising repurposed drug candidates; and (3) the algorithm is able to scour and map patterns at a pace and breadth that exceeds human domain expertise. For example, SemNet was able to identify novel repurposed drugs for COVID-19 [11], and approximately 40% were later clinically validated as positive adjuvant therapies [12].

The key contributions of this investigation are as follows: The results of this study prioritize repurposed drug candidates that may synergistically improve symptom management in PD when used with standard-of-care dopamine therapies, like levodopa and its derivatives. The investigation utilizes an innovative cross-domain text-mining approach that examines a large knowledge graph of 33+ million journal articles to identify the best repurposed drug candidates. Unlike a manual systematic review or meta-analysis, this artificial intelligence approach enables a truly comprehensive evaluation of the literature across multiple domains to identify and compare potential repurposed drug candidates for PD. The key clinical finding of this study was that antihistamines were ranked as the most promising repurposed pharmacological drug for PD by artificial intelligence. We present the results of the cross-domain text-mining analysis and provide evidence as to how antihistamines could potentially improve PD treatment by decreasing oxidative stress, decreasing inflammation, and improving neurotransmitter imbalance.

## 2. Results

**Figure 1:**
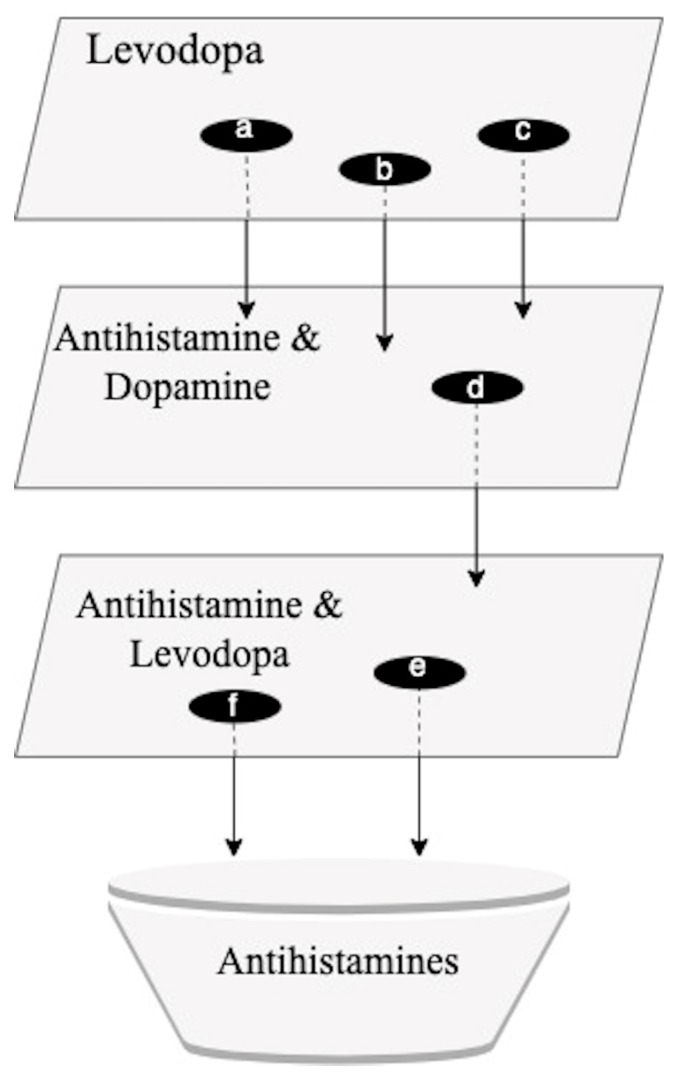
Sequence of simulation searches in SemNet. For each simulation layer, specific “hub nodes” were identified. Hub nodes have a predicted strong relationship (e.g., HeteSim score) with the target node or query. The identified hub nodes were subsequently used as targets for the next layer of searches. The synthesis of information from all the SemNet 2.0 simulation layers contributed to the identification of antihistamines as a promising group of repurposed PD drugs. a = piroxicam; b = leflunomide; c = loratadine; d = ebastine; e = levocetirizine; f = ebastine.

A series of multiple SemNet 2.0 simulations were required to perform a comprehensive cross-domain text-mining analysis necessary to identify promising repurposed drug candidates for PD. SemNet 2.0 [8] was utilized to identify drugs that showed a strong relationship with PD and related standard-of-care drugs like levodopa. A series of simulation searches, as shown in Figure 1, was performed using a technique called hub analysis. Hub analysis assisted in the identification of cross-domain relationships to repurposed drug candidates that have an important but less obvious connection to PD. Initial simulations were performed with the target node set to “Levodopa” (Figure 1). The initial simulation layer yielded a total of 1890 source node results. The hubs identified from one simulation became targets for the next simulation, as depicted in Figure 1. The serial layers and identification of hubs enabled less obvious or more distant cross-domain relationships to be included in the ranking algorithm. The ranking of promising repurposed drug candidates was based on (1) the resultant SemNet 2.0 HeteSim scores and (2) the recurrence of a high-ranking source node across multiple search layers.

### 2.1. Antihistamines Are a Category of Drugs Strongly Related to PD

**Figure 2:**
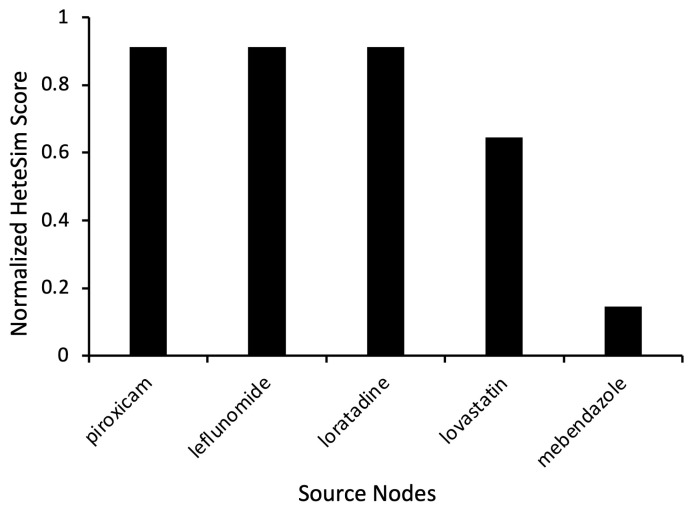
Representative source node results from SemNet 2.0 simulations with the target node “Levodopa”. The source node type used was “Clinical Drug” (CLND). HeteSim scores were normalized to enable comparison of nodes across multiple simulations.

As evidenced by relevant HeteSim scores of 0.911, the results most notably show piroxicam, leflunomide, and loratadine as having comparatively high levels of relatedness to levodopa (Figure 2). Piroxicam is a non-steroidal anti-inflammatory drug (NSAID) commonly used to treat rheumatic diseases [13]. Similarly, leflunomide is a disease-modifying antirheumatic drug (DMARD) used to treat rheumatoid arthritis. The medication has anti-inflammatory and immunoregulatory properties [14]. Loratadine is a second-generation antihistamine commonly used to treat allergies [15]. Less related simulation results included lovastatin, a medication used to treat high cholesterol. It yielded a HeteSim score of 0.645, while mebendazole, an anthelmintic medication, had a HeteSim score of 0.146 (Figure 2).

**Figure 3:**
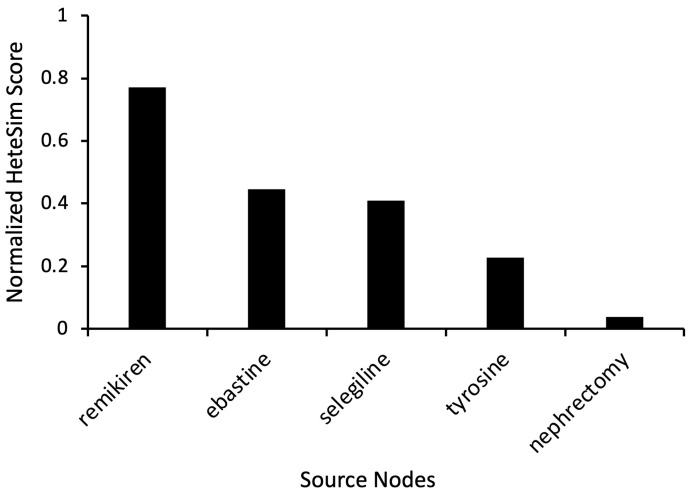
Representative source node results from SemNet 2.0 simulations with target nodes of “Antihistamine” and “Dopamine”. The source node types were “Clinical Drug” (CLND), “Pharmacologic Substance” (PHSU), and “Therapeutic or Preventative Procedure” (TOPP). HeteSim scores were normalized to enable comparison of nodes across multiple simulations.

To further investigate the antihistamine drug type and explore non-conventional drugs predicted to modulate dopamine, additional simulations were performed with the target nodes as “Dopamine” and “Antihistamine”, as shown in Figure 1. The number of Unified Medical Language System (UMLS) source node types used was increased to expand the scope of the search. Instead of only using the category “Clinical Drug” (CLND), the source node types “Pharmacologic Substance” (PHSU) and “Therapeutic or Preventative Procedure” (TOPP) were also added. These simulations yielded results from drug categories like renin inhibitors (remikiren), antihistamines (ebastine), and antidepressants (selegiline). Ebastine had a HeteSim score of 0.447 (Figure 3).

### 2.2. Levocetirizine and Ebastine Are Predicted as Promising Repurposed Drugs for PD

**Figure 4:**
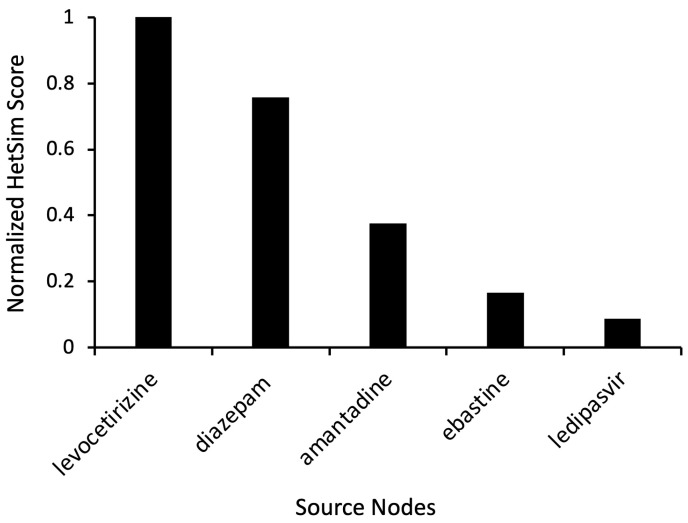
Representative source node results from SemNet 2.0 simulation with target nodes of “Antihistamine” and “Levodopa.” The source node type was “Clinical Drug” (CLND). HeteSim scores were normalized to enable comparison of nodes across multiple simulations. Notice that ebastine reoccurs as a source node.

Based on the preliminary simulation results and corresponding HeteSim scores, additional simulations were run with the target nodes set to “Antihistamine” and “Levodopa” (Figure 1). Levocetirizine displayed a high HeteSim relatedness score of 0.999, and the HeteSim score for ebastine was 0.166 (Figure 4). The notable appearance and reappearance of antihistamines like levocetirizine and ebastine, respectively, suggested the need for further analysis of these specific drugs. As shown in Figure 4, other simulation results included anxiolytics (diazepam), dopamine agonists (amantadine), and drugs used to treat hepatitis C (ledipasvir).

### 2.3. Fourteen Highly Ranked Source Nodes Were Selected as Hubs for Further Analysis

**Figure 5:**
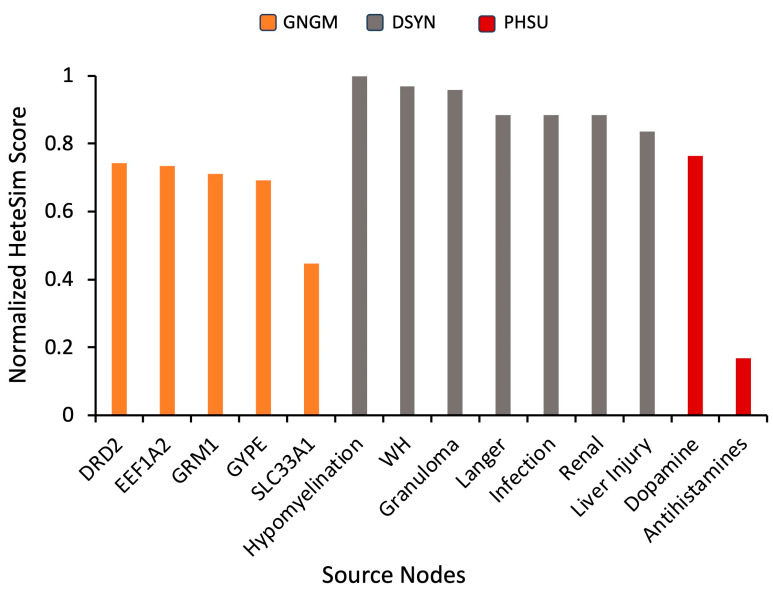
Normalized HeteSim scores of returned source nodes selected as hubs using “Parkinson’s Disease” as a SemNet 2.0 simulation target node. HeteSim scores were normalized to enable comparison of nodes across multiple simulations. Color code represents source node type: GNGM (gene or genome), DSYN (disease or syndrome), or PHSU (pharmacologic substance). Descriptions of shown source nodes: “Hypomyelination” is hypomyelination within brainstem and spinal cord; “WH” is Werdnig Hoffmann paralysis; “Granuloma” is granuloma of intestine; “Langer” is Langer mesomelic dysplasia syndrome; “Infection” is infection in the elderly; “Renal” is high renal threshold for glucose; “Liver injury” is drug-induced liver injury.

In order to identify highly connected hub nodes of significance, a supplemental simulation was run using “Parkinson’s disease” as the target node. The UMLS node types were assigned to be “Disease or Syndrome” (DSYN), “Gene and Genome” (GNGM), or pharmacologic substance (PHSU). Using the hub analysis process detailed in Section 4.3, source nodes with highly ranked HeteSim scores were selected for further analysis from the simulation results (Figure 5). The 14 source nodes included DRD2, EEF1A2, GRM1, GYPE, SLC33A1, hypomyelination, WH (Werdnig Hoffmann paralysis), granuloma (granuloma of intestine), Langer (Langer mesomelic dysplasia syndrome), infection (infection in the elderly), renal (high renal threshold for glucose), liver injury, dopamine, and antihistamines. Note that dopamine and antihistamines were recurring source nodes.

### 2.4. Four Additional Drugs Were Discovered by Hub Analysis

Each of the 14 hub nodes were then designated as the target node for 14 additional individual simulations. The UMLS node types were “Pharmacologic Substance” (PHSU), “Amino Acid, Peptide, or Protein” (AAPP), and “Disease or Syndrome” (DSYN). Cross evaluation of the simulation results from all the hub nodes led to the identification of specific drugs that were highly ranked across multiple simulations. The antihistamine ebastine was used to back-evaluate the hubs. Ebastine was chosen for back-evaluation because it was highly ranked in all simulation layers. After further investigation and validation of SemNet 2.0 rankings by manual inspection of the full text of the relevant biomedical literature, several drugs were deemed to be promising (Table 1). The four drugs most related to the 14 hub nodes were vancomycin, captopril, neramexane, levocetirizine, and ebastine. As such, these four source nodes represent repurposed drugs that machine learning predicts are the most promising repurposed adjuvant therapies for PD.

## 3. Discussion

Cross-domain text mining of biomedical relationships [10] is a cutting-edge artificial intelligence technique to identify and rank repurposed drugs that could serve as adjuvant therapies for presently intractable diseases, including PD. Advanced artificial intelligence techniques harness the power of NLP and machine learning to rank promising literature relationships in an unsupervised and less biased manner. The overall results predicted ebastine, levocetirizine, vancomycin, captopril, and neramexane as repurposed drug candidates that are most likely to be beneficial to PD. That is, these repurposed drugs represent promising adjuvant therapies that could improve the impact of current standard-of-care PD therapies like levodopa and levodopa–carbidopa.

The cross-domain text mining of relationships from 33+ million PubMed articles determined ebastine and levocetirizine to be among the highest-ranked repurposed pharmacologic substances (Figure 2). Namely, ebastine had a connection to both antihistamines and dopamine (Figure 3). Ebastine and levocetirizine are antihistamines primarily utilized to treat perennial allergic rhinitis (e.g., seasonal allergies) [16]. While antihistamines are not yet clinically proven for PD, there is evidence of their positive effect. In fact, one study suggested that, when used with levodopa, ebastine and levocetirizine each significantly improved PD-like side effects induced in mouse models. The benefit was further amplified when the treatments were administered in combination with levodopa, and the mRNA expression of PD markers was significantly lowered [17]. These preclinical findings show the potential value of co-administering antihistamines with levodopa to better manage the motor hallmarks of clinical PD.

Presently, there are four known categories of histamine receptors. These include the H1, H2, H3, and H4 receptor types [18]. The most common antihistamines target the H1 and H2 receptors; however, antihistamine-like drugs inhibiting the effects of histamine at H4 and H3 receptors have also been developed [19]. Several of the histamine receptor types play a role in PD progression, and the brains of individuals with PD display dysregulation and abnormal distribution of histamine receptors [20,21]. Through H1 and H4 receptor activation, histamine facilitates reactive oxygen species (ROS) production as well as microglial phagocytosis. These inflammatory mechanisms contribute to dopaminergic neurotoxicity and cell death implicated in PD [22].

Considering the importance of histaminergic activity in PD pathology, the modulation of histamine via antihistamines represents a promising therapeutic focus. The blockage of H1 receptors safeguards against histamine-induced dopaminergic neuron death in vivo [22]. Additionally, H2 receptor antagonists improve PD motor symptoms and protect against apoptosis in dopaminergic cells [23].

In rotenone-induced PD rat models, H4 receptor antagonists normalize dopamine levels [24]. In MPTP-induced PD mice models, the inhibition of H3 receptors demonstrates neuroprotective effects and reduces PD pathophysiology [20].

Interestingly, antihistamines were previously found to be a positive adjuvant therapy for another neurodegenerative disease, Amyotrophic Lateral Sclerosis (ALS) [25]. The hypothesized mechanism of action for their benefit in clinical ALS was initially thought to be related to airway secretion clearance [25]. However, it is possible that antihistamines have several of the same positive neuroprotective effects for ALS as they do in PD—namely, combatting oxidative stress and decreasing inflammation.

### 3.1. Oxidative Stress in PD

Oxidative stress exacerbates neurodegeneration in PD [26]. PD-causing gene products like alpha-synuclein, parkin, PINK-1, LRRK2, and DJ-1 increase the production of reactive oxygen species (ROS) and free radicals [27]. As such, PD patients have a greater susceptibility to the effects of oxidative stress. A vicious cycle occurs as initial oxidative-stress-related damages result in further insults to prominent pathogenic proteins that, in turn, induce additional ROS production. The unfavorable modifications of molecular pathways caused by compounding oxidative stress ultimately result in neuronal death. One potential way to mitigate the effects of oxidative stress is to utilize antioxidants. Antioxidants play an essential role in defending the body against oxidative damage. Specifically, antioxidants prevent the formation of free radicals and can neutralize their effects [28]. In healthy individuals, protection against oxidative harm is maintained by a balance between oxidants and antioxidants [29]. Facilitating the production of antioxidants helps restore ROS homeostasis, which may decrease ROS-enhanced neurodegeneration.

Ebastine was one of the highly ranked repurposed drugs predicted by cross-domain text mining. Ebastine induces a dose-dependent increase in the activity of antioxidant enzymes like SOD, CAT, and GSH as well as a decrease in accompanying biomarkers of oxidative stress like MDA and nitrite [17]. Additionally, levocetirizine, another highly ranked cross-domain text-mining result, similarly fosters a significant increase in the activity of SOD [30]. The ability of ebastine and levocetirizine to increase the levels of antioxidant enzymes suggests that these antihistamines may help ameliorate neurodegeneration by increasing the number of compounds that can counteract oxidation. As such, ebastine and levocetirizine may help neutralize the PD-inducing insults caused by free radicals and ROS.

### 3.2. Neurotransmitter Imbalance in PD

Due to the degeneration of dopaminergic neurons in the midbrain, the concentrations of dopamine are reduced [31]. This dopamine deficiency disrupts the balance between dopamine and acetylcholine as the level of acetylcholine begins to surpass that of dopamine [32]. Adequate and appropriately balanced concentrations of both neurotransmitters are required to maintain proper motor function; therefore, with skewed levels, PD motor symptoms are unable to return to normal. In addition to decreased dopamine, imaging has also illustrated decreased noradrenergic and serotonergic transmission in the brains of PD patients [33]. The use of antihistamines may be able to re-establish suitable levels of these neurotransmitters and improve related Parkinsonian symptoms. Ebastine and levocetirizine have been experimentally shown to increase the levels of dopamine, serotonin, and noradrenaline to improve the motor and non-motor features of PD. The reinstatement of a proper balance between dopamine and acetylcholine is another possible mechanism of action for ebastine and levocetirizine to improve PD. Ebastine and levocetirizine have been found to decrease acetylcholine levels in mouse models [17]. The alleviation of neurotransmitter imbalances indicates the potential utility of antihistamines as a repurposed drug or adjuvant therapy for PD.

### 3.3. Inflammation in PD

Chronic inflammation is regarded as a pronounced agitator in PD due to its role in causing neurotoxicity and cell death [34]. The concomitant dysregulation of inflammatory mediators is a related aggravator [35]. In PD patients, the production and activity of inflammatory mediators like histamine and TNF-α are upregulated. Abnormally heightened brain concentrations of histamine and increased density of histaminergic fibers in the substantia nigra of PD patients likely contribute to progressive dopaminergic neuron death [36,37,38]. Elevated TNF-α cytokine levels found in the blood, cerebrospinal fluid, and brain of PD patients have also been implicated in the progression of the disease due to the molecule’s role in inciting inflammatory activity and cell apoptosis [39,40]. Modulating the supply and activity of inflammatory regulators may protect against PD-related neural pathology [1,20]. Antihistamines may improve neurodegeneration by countering the inflated concentrations of inflammatory regulators. Compared to the excess amounts of histamine and TNF-α in untreated haloperidol-induced PD animal models, animals treated with ebastine and levocetirizine demonstrated a dose-dependent decrease in the levels of inflammatory molecules [17]. The observed lowering of histamine and TNF-α content following the administration of ebastine and levocetirizine reflects the potential value of using antihistamines in combatting inflammation-induced progression in PD.

Beyond antihistamines, cross-domain text mining with hub analysis identified strong relationships to vancomycin, captopril, and neramexane, which also have anti-inflammatory properties. (Table 1). Previous studies have identified the utility of anti-inflammatory drugs in PD [41,42]. Captopril, a vasodilator with anti-inflammatory effects, downregulates the angiotensin II system by blocking the activity of angiotensin-converting enzyme (ACE) [43]. Angiotensin II plays a prominent role in the degeneration of dopaminergic neurons via activation of the AT1 receptor [44]. Vancomycin, a glycopeptide antibiotic with anti-inflammatory effects, inhibits monoamine oxidase B (MAO-B), which is an enzyme that metabolizes dopamine [45]. Due to its antibacterial nature, vancomycin alters gut microbiota and fecal short-chain fatty acid levels [46]. This, in turn, can decrease the expression of the TLR4/MyD88/NF-κB/TNF-α signaling pathway in the brain and the gut. Through the suppression of this signaling pathway, the activities of astrocytes and microglia are limited, which mitigates worsening inflammation [45]. With the inhibition of MAO-B by vancomycin in PD patients, dopamine levels can be increased and maintained at appropriate levels [47]. Neramexane, an NMDA antagonist with neuroprotective and anti-inflammatory properties, counteracts induced decreases in dopamine levels in the substantia nigra pars compacta of rats [48]. The antagonistic activity of this drug on NMDA receptors protects dopaminergic neurons in the striatum and substantia nigra pars compacta from neurotoxicity that could eventually lead to PD-related oxidative stress and inflammation [48].

### 3.4. Possible Role of Liver in PD

As shown in the results, several of the identified source nodes had ties to liver disease or injury (Figure 4 and Figure 5, Table 1). Liver-related drugs or treatments also repeatedly appeared over multiple simulations. In fact, ledipasvir, a drug used to treat hepatitis, was one of the key nodes found as part of the antihistamine hub node analysis (Figure 4). The mechanistic connection to liver disease is not entirely clear but is nonetheless interesting. A study of 120 patients with liver cirrhosis found that 52% of participants also displayed signs of PD [49]. Additionally, a retrospective cohort study of various types of hepatitis reported increased rates of subsequent PD in individuals with hepatitis B and hepatitis C [50]. Growing evidence suggests that the prevalence of liver disease history among PD patients is connected to the inability of the liver to clear the blood of neurotoxins like α-syn and manganese (Mn), which can then accumulate in the liver [51,52]. Compromised hepatic ability resulting from liver disease may sabotage the removal of pathological proteins, allowing neurotoxins to enter cerebral circulation and facilitate PD pathogenesis [52]. The entrance of toxic substances into the brain is further provoked by portosystemic shunting, which often occurs as a result of advanced liver diseases like acquired hepatocerebral degeneration (ADH) [52]. Portosystemic shunting has been implicated in the accrual of Mn in the basal ganglia, leading to the appearance of Parkinsonian symptoms [49]. Other hypotheses suggest liver infections like hepatitis C release inflammatory cytokines, which can promote the progression of PD [53]. Ties to liver disease have also been suggested in other neurodegenerative diseases, including ALS [54] and, more recently, Alzheimer’s Disease [55].

### 3.5. Limitations for Clinical Implementation of Antihistamines for PD

As a note of importance, the feasibility of using some machine-learning-identified repurposed drugs for PD treatment may vary. In particular, unlike levocetirizine and captopril, ebastine has not been approved by the Food and Drug Administration in the United States of America. However, ebastine has been approved in other countries. At the time of this writing, the use of neramexane is unlikely due to its clinical mass production being presently discontinued. Vancomycin may also be a less feasible option considering the risk of antibiotic resistance. Thus, not all the identified promising drugs in this study can be immediately clinically utilized at this time. Nonetheless, their identification is helpful for elucidating the mechanisms of action that could be leveraged in future drug development for PD.

Another concern is the reduced effectiveness of second-generation antihistamines due to the limited ability of such drugs to cross the blood–brain barrier. Compared to first-generation antihistamines, second-generation H1 antihistamines translocate across the blood–brain barrier to a lesser extent [56]. In order to address this, further studies regarding drug delivery at the blood–brain barrier should be conducted. Strategies may include the use of carrier mechanisms or the enhancement of lipid solubility [57] or direct administration using intrathecal drug delivery.

The potential side effects of antihistamines may warrant questions about their utility compared to, or in addition with, other commercially available medications, namely levodopa modulators or replacements. Beyond the “wearing off” effect of levodopa, the common side effects of levodopa–carbidopa include dizziness, loss of appetite, nausea, diarrhea, dry mouth, mouth and throat pain, constipation, change in taste, forgetfulness or confusion, anxiety, nightmares, insomnia, headache and weakness, hoarseness, dyskinesias, and rapid heart rate [58]. Some of the more common side effects with antihistamines include drowsiness, dry mouth, blurred vision, dizziness, headache, low blood pressure, mucous thickening, rapid heart rate, and difficulty urinating [59]. Notably, there is some overlap in the side effects of levodopa–carbidopa and antihistamines that would require further assessment and careful dose titration. As previously shown, diet and vitamin therapy may be one way to offset the side effects of Parkinsonian therapies [60].

Despite the possibility of side effects, the potential benefit of incorporating antihistamines into PD treatment is that antihistamines address multiple facets of the PD pathology, including oxidative stress, inflammation, and neurotransmitter imbalance. Moreover, despite the value of levodopa in rectifying the depletion of dopamine, levodopa treatment has also been reported to aggravate PD progression by fostering the release of inflammatory cytokines [61]. Therefore, the possible advantages of incorporating antihistamines as part of a comprehensive PD treatment should not be overlooked. In summary, antihistamines are not a replacement for dopamine therapies. However, antihistamines are a possible adjuvant therapy that could potentially address the multi-factorial nature of PD pathology beyond the lack of dopamine.

### 3.6. Investigation Limitations and Future Directions

This investigation utilized cross-domain text mining of 33+ million journal articles in PubMed and artificial intelligence methods to rank repurposed drug candidates that have the most potential as adjuvant PD therapies. A key limitation of this study is the identification of relationships from the existing literature in PubMed. While the SemNet technology has been shown to identify the majority of relationships from the literature, infrequent or differently worded relationships may not be transformed properly into the knowledge graph [8,9]. Additionally, the investigation can only identify already published relationships and integrate them across domains into a comprehensive knowledge graph. The unsupervised learning ranking algorithm has corrections (e.g., such as a degree weighted path count) to ensure that relationships with a lower count or few citations are not completely overlooked in the HeteSim importance rankings [8]. However, there is still a possibility that newer or less cited work may be under-ranked. Finally, there is no correction for data source quality in the knowledge graph. Rather, all data sources in the knowledge graph are treated equally rather than being weighted by a quality index, impact factor, or citation rate.

Future work in cross-domain text mining should address the integration of multiple different databases (beyond PubMed), improved semantic extraction of relationships for integration into the knowledge graph, and the ability to assess perceived data source quality [8]. Additionally, future work may be able to use existing relationships from the literature to predict unreported, novel relationships that have yet to be published. One such method is link prediction. For example, link prediction was used in conjunction with SemNet technology to identify novel relationships for the treatment of COVID-19 [11]. In addition to drug repurposing, link prediction models may also aid in the development of novel drug targets and formulations.

## 4. Materials and Methods

### 4.1. Overview of SemNet 2.0

SemNet 2.0 is a text-mining tool that optimizes literature-based discovery within an interactive Python-based framework [8]. Compared to its predecessor, SemNet version 1 [62], SemNet 2.0 uses a much faster processing speed to query the National Library of Medicine’s SemMedDB repository to create a knowledge graph, composed of numerous nodes and edges, that identifies relationships among biomedical concepts. Nodes correspond to a United Medical Language System (UMLS) biomedical concept (PD, COVID-19, etc.) along with an associated semantic type (therapeutic or preventative procedure, disease or syndrome, etc.). This study differentiates its target and sources by referencing them as a “target node” and “sources-node”. An edge refers to a connection between concepts, a target and source, that encodes a UMLS prediction (treats, inhibits, causes, etc.) [8].

With natural language processing and machine learning techniques, SemNet 2.0 can manipulate the knowledge graph by constraining it based on UMLS node type (pharmacologic substance, pathological function, therapeutic or preventative procedure, etc.) and relation type (inhibits, teats, affects, etc.). Therefore, metapaths present more related source nodes. A metapath connects the user-specified target node to the related source node through a series of sequential node and relationship types in the graph. These metapaths are used to calculate HeteSim scores. This score lies within the interval (0.1) and is a normalized measure of relatedness between concept nodes that considers all metapaths (e.g., the paths that connect nodes of interest) [8]. Each simulation uses the standard unsupervised learning rank aggregation algorithm in SemNet 2.0 to examine published relationships in the constructed knowledge graph. For the current study, a total of 19 simulations were performed.

**Figure 6:**
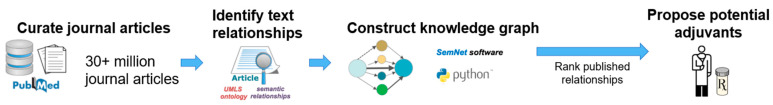
Overview of the cross-domain text-mining method. Over 33+ million journal articles from PubMed are text mined. Relationships are extracted according to the Unified Medical Language System (UMLS) ontology to construct a large-scale knowledge graph in a recently developed cross-domain text-mining software called SemNet 2.0 [8]. Artificial intelligence methods mine relationship patterns to identify promising candidates using “levodopa” and “Parkinson’s Disease” as the primary target nodes for the initial series of searches. Specifically, unsupervised learning rank aggregation assigned a ranking to filter the most promising repurposed drugs for PD.

**Figure 7:**
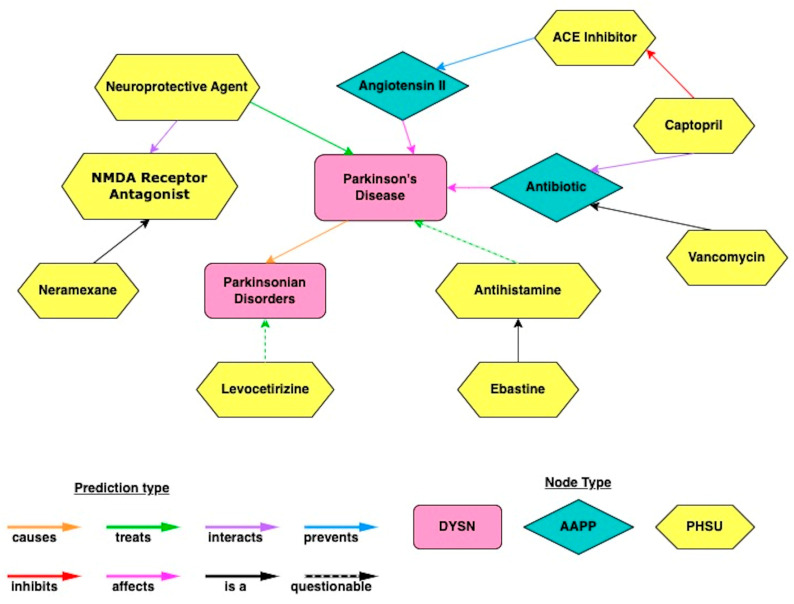
An example subgraph (>99.99% pruned) obtained by querying the large SemMedDB knowledge graph using cross-domain text mining in SemNet 2.0. UMLS node types included are “Pharmacologic Substance” (PHSU), “Amino Acid, Peptide, or Protein” (AAPP), and “Disease or Syndrome” (DSYN). Note the full, unpruned graph is too large to visualize and would be intractable to the human eye.

Figure 6 illustrates the overview of the cross-domain text-mining method. Data were extracted from the text of journal articles contained within the PubMed database. Then, text relationships were identified as subject–object–predicate triples, which connect two nodes together. A collection of relationship triples constitutes a knowledge graph. By ranking such relationships, potential repurposed drugs for PD were identified. Figure 7 depicts an example subgraph from the larger knowledge graph. The visualized subgraph is highly pruned (>99.9% pruned). The full knowledge graph cannot be shown as it is too large and intractable to visualize with the human eye. The shapes in Figure 7 represent nodes, which are biomedical concepts found in the literature as defined by the UMLS ontology. Example node types included are “Pharmacologic Substance” (PHSU), “Amino Acid, Peptide, or Protein” (AAPP), and “Disease or Syndrome” (DSYN). Finally, cross-domain text-mining analysis to identify repurposed drug candidates for PD was performed using a form of hub node network analysis we previously described [10]. Hub node network analysis enables more distant cross-domain relationships in the knowledge graph to be fairly represented in the resultant drug candidate rankings.

SemNet 2.0 utilizes unsupervised learning rank aggregation to rank the most important nodes by examining metapath patterns within the knowledge graph. HeteSim scores provide a predicted importance ranking with respect to the user-defined target node(s). The mathematical derivation and calculation of the HeteSim score and its specific algorithmic implementation in SemNet 2.0 has been previously described [8]. HeteSim scores are normalized between simulations and used to compile an aggregated list of the most promising repurposed drug candidates for PD. In the present work, HeteSim scores vary between 0 and 1, with scores closer to 1 representing higher ranked nodes the algorithm deems as “more relevant” or “more important” to the specified target nodes.

### 4.2. Preliminary Simulations

An initial SemNet 2.0 [8] simulation was performed with the target node as “Levodopa”. Since it is widely used as a current standard-of-care PD therapy, levodopa was specified as the first target node to start searching for potential related source nodes that could suggest additional drugs that may be relevant to PD. Based on HeteSim scores, the most promising identified source nodes were predominantly anti-inflammatory drugs and antihistamines. As such, simulations were subsequently constrained to find connections between inflammation, antihistamines, dopamine, and levodopa. Table 2 details the specific simulations run with target nodes and node types. Node types are based on the UMLS ontology.

### 4.3. Hub Analysis

Hub analysis is frequently used in bioinformatics to trace downstream gene relationships to hub genes and analyze complicated networks [10,62,63,64]. Much like a wheel where spokes converge to the wheel hub, a hub node is a node that has a large degree of connected, related nodes in the network [65]. Analogous to hub network analysis in bioinformatics, hub analysis [10] can be performed on a knowledge graph to look at relationships that are further downstream from the user-defined target of interest. This is accomplished by examining relationships around a set of selected “hub nodes” that are connected to the target of interest. Hub nodes were selected to explore similar drugs that have less exposure in the treatment of PD. As shown in the preliminary results, dopamine, anti-inflammatory drugs, and antihistamines were the returned highest-ranking source nodes and, thus, were chosen as hubs for further analysis.

Genetic connections in the pathology and mechanisms of similar diseases can be exploited to find less-studied drugs. Thus, diseases and genes were also explored as hubs. To find the specific nodes that can be used as hubs, simulations were run with “Parkinson’s disease” as the target and “Disease or Syndrome” (DSYN) and “Gene and Genome” (GNGM) as UMLS node types. These results are displayed in Figure 5.

After hubs were selected, a simulation was run on each hub using the following UMLS node types: “Pharmacologic Substance” (PHSU), “Amino Acid, Peptide, or Protein” (AAPP), and “Disease or Syndrome” (DSYN). These node types were chosen to better filter specific substances that may be useful. A back-evaluation of the hubs was conducted to evaluate their efficacy. The back-evaluation assessed whether the most promising drug identified from the preliminary simulations, ebastine, was reproduced within the highly ranked candidates from hub analysis. Identification of candidates within both the preliminary simulations and the hub analysis provided additional credence for their inclusion in the final repurposed drug recommendation list. Finally, a cross-evaluation was conducted to find nodes with relevant HeteSim scores across multiple simulations. HeteSim scores were then compiled to determine the final list of predicted promising PD drug candidates and their likely mechanisms of action for ameliorating the PD pathology.

## 5. Conclusions

The goal of this study was to utilize innovative text mining and artificial intelligence algorithms to suggest helpful repurposed drugs as adjuvant therapies that may improve PD treatment in combination with standard-of-care drugs like levodopa–carbidopa. Cross-domain text mining and hub analysis were performed with SemNet 2.0 using a knowledge graph of relationships extracted from 33+ million PubMed journal articles. Machine learning predicted the best repurposed drug candidates to potentially serve as positive adjuvant therapies for PD: ebastine, levocetirizine, vancomycin, captopril, and neramexane. Not all the aforementioned drugs may be clinically feasible at this time. Nonetheless, the identification of these drugs as promising to PD provides insight into underlying mechanisms that could be exploited in future PD drug development. Antihistamines were among the highest-ranked repurposed therapies predicted by artificial intelligence. In particular, cross-domain text-mining analysis suggests that antihistamines provide protection against neurodegeneration by ameliorating oxidative stress, improving neurotransmitter balance, and decreasing inflammation. In summary, antihistamines may synergistically alleviate Parkinsonian symptoms when used with dopamine modulators like levodopa or levodopa–carbidopa. Therefore, antihistamines warrant further experimental investigation as a potential future adjuvant therapy for PD.

## Figures and Tables

**Table 1:** Summarized results of hub analysis. The four drugs validated as most related to the 14 hub nodes shown in Figure 5 were vancomycin, captopril, neramexane, levocetirizine, and ebastine. Thus, the hub analysis results are organized into columns associated with either vancomycin, captopril, neramexane, levocetirizine, or ebastine. The respective normalized HeteSim scores for each hub node SemNet 2.0 simulation are listed in descending order. Only hubs with normalized HeteSim scores greater than 0.2 are shown. The results collectively represent additional cross-domain source nodes predicted by the algorithm as relevant to Parkinson’s Disease.

Vancomycin	Captopril	Neramexane	Levocetirizine	Ebastine *
Granuloma (0.927)	Hypomyelination (0.474)	SLC33A1 (0.765)	EEF1A2 (0.455)	SLC33A1 (0.585)
Liver Injury (0.499)	Dopamine (0.462)	Antihistamine (0.706)	Dopamine (0.391)	Antihistamine (0.446)
Renal (0.341)	Renal (0.436)	EEF1A2 (0.629)	DRD2 (0.375)	DRD2 (0.399)
Hypomyelination (0.333)	SLC33A1 (0.335)	DRD2 (0.505)	SLC33A1 (0.316)	EEF1A2 (0.398)
SLC33A1 (0.272)	GRM1 (0.271)	GRM1 (0.492)		GRM1 (0.369)
GYPE (0.265)	GYPE (0.249)	GYPE (0.408)		
DRD2 (0.251)	EEF1A2 (0.247)			
EEF1A2 (0.245)	DRD2 (0.237)			

* Ebastine was used as back-evaluation of the hubs.

**Table 2:** A list of preliminary SemNet 2.0 simulations used in the current study. The objective column briefly explains why the simulation was performed. The target node with CUI column specifies the biomedical concept(s) utilized as user-specified target node(s) in SemNet 2.0, along with their corresponding UMLS concept unique identifier (CUI). The node types column specifies the UMLS node types examined: CLND: “Clinical Drug”, TOPP: “Therapeutic or Preventive Procedure”, PHSU: “Pharmacologic Substance”.

Simulation	Objective	Target Node with CUIs	Node Types
1	Explored connections to levodopa that may be an uncommon relation to PD.	levodopa (C0023570)	CLND
2	Examined anti-inflammatory properties of drugs/substances relevant to dopamine.	inflammation (C0021368), dopamine (C0013030)	CLND, TOPP, PHSU
3	Examined relations between antihistamines and dopamine.	antihistamine (C3536809), dopamine (C0013030)	CLND, TOPP, PHSU
4	Explored potential antihistamine adjuvants with properties related to levodopa.	antihistamine (C3536809), levodopa (C0023570)	CLND, TOPP, PHSU
5	Examined potential relevance of antihistamines to tremor, a common PD symptom.	antihistamine (C3536809), tremor (C0023570)	CLND, TOPP, PHSU

## Data Availability

SemNet 2.0 code can be found on GitHub https://github.com/pathologydynamics/semnet-2 (accessed on 1 August 2023).
